# A Network-Pharmacology-Combined Integrated Pharmacokinetic Strategy to Investigate the Mechanism of Potential Liver Injury due to *Polygonum multiflorum*

**DOI:** 10.3390/molecules27238592

**Published:** 2022-12-06

**Authors:** Zhixin Jia, Lirong Liu, Cong Fang, Mingxia Pan, Shiyu Cong, Zhonghui Guo, Xiaoqin Yang, Jie Liu, Yueting Li, Hongbin Xiao

**Affiliations:** 1 Beijing Research Institute of Chinese Medicine, Beijing University of Chinese Medicine, Beijing 102401, China; 2 Research Center of Chinese Medicine Analysis and Transformation, Beijing University of Chinese Medicine, Beijing 102401, China; 3 School of Chinese Materia Medical, Beijing University of Chinese Medicine, Beijing 102401, China

**Keywords:** *Polygonum multiflorum* (PM), integrated pharmacokinetics, network pharmacology, potential liver injury

## Abstract

*Polygonum multiflorum* (PM) has been used as a tonic and anti-aging remedy for centuries in Asian countries. However, its application in the clinic has been hindered by its potential to cause liver injury and the lack of investigations into this mechanism. Here, we established a strategy using a network pharmacological technique combined with integrated pharmacokinetics to provide an applicable approach for addressing this issue. A fast and sensitive HPLC-QQQ-MS method was developed for the simultaneous quantification of five effective compounds (trans-2,3,5,4′-tetrahydroxystilbene-2-*O*-β-d-glucoside, emodin-8-*O*-β-d-glucoside, physcion-8-*O*-β-d-glucoside, aloe-emodin and emodin). The method was fully validated in terms of specificity, linearity, accuracy, precision, extraction recovery, matrix effects, and stability. The lower limits of quantification were 0.125–0.500 ng/mL. This well-validated method was successfully applied to an integrated pharmacokinetic study of PM extract in rats. The network pharmacological technique was used to evaluate the potential liver injury due to the five absorbed components. Through pathway enrichment analysis, it was found that potential liver injury is primarily associated with PI3K-Akt, MAPK, Rap1, and Ras signaling pathways. In brief, the combined strategy might be valuable in revealing the mechanism of potential liver injury due to PM.

## 1. Introduction

*Polygonum multiflorum* (PM) originates from the root of *Polygonum multiflorum* Thunb, a traditional Chinese medicine (TCM) plant that belongs to the polygonaceae plant family [1]. PM contains various compounds, such as stilbenes, anthraquinones, flavonoids, lecithin, tannin, and trace elements, among which the stilbenes and anthraquinones are considered to be the mainly active or potentially toxic components [2,3,4,5]. Raw PM is mainly used for eliminating carbuncles, preventing malaria, detoxification, and relaxing the bowel, whereas processed PM is used as a tonic and for immune enhancement [6]. During the past decades, PM has become popular because of the growing interest of the general population in alternative medicines and phytonutrients. However, an increasing number of reports the adverse hepatic effect of PM or proprietary Chinese medicinal products containing PM have been received since the 1990s worldwide [7,8]. Usually, long-term usage or large doses of PM are considered to cause liver injury [9], but some researchers think that the potential liver injury associated with PM is idiosyncratic and is not related to the dose, duration, or route of drug administration [10,11]. Therefore, the findings mentioned earlier necessitate further study that aims to carry out a deeper investigation into the underlying mechanism of the hepatotoxicity of PM.

The effective/toxic components absorbed into the body are the key to the effects of the efficacy/toxicity of TCM. The pharmacokinetic characteristics of these active ingredients have been used to predict the efficacy and potential toxicity of TCM and provide guidance for the rational clinical usage of drugs [12,13]. Thus, the investigation of the metabolic processes of effective components in vivo by pharmacokinetics is of great importance and could provide scientific data support and a reference for clarifying the pharmacological or toxicological action of TCM. Usually, pharmacokinetic work mostly focuses on a single component or some isolated components [14,15], rather than considering them as a whole, which is not in accordance with the characteristics of the multiple components and multiple targets of TCM. In recent years, integrated pharmacokinetic studies have been proposed; this was first reported by Wang et al. [16] based on the “area under the curve (AUC) weighting integrated” method. In this method, the integrated pharmacokinetic parameters of multiple components in vivo replaced the parameters of single compounds, which could reveal the pharmacokinetic characteristics of multiple components more comprehensively and systematically [17,18,19]. To date, there have been some reports on the pharmacokinetic studies of PM [20,21,22], mainly focused on its single component, investigating pharmacokinetics, tissue distribution, and excretion. However, no reports regarding the integrated pharmacokinetic study of the multiple components of PM have been carried out so far, and no studies have explored the underlying mechanism of hepatotoxicity based on pharmacokinetics.

Network pharmacology explains drug action and its mechanism based on a network of interactions between drugs, targets, and diseases. The research mode of a “multi-component, network target effect” is in line with the characteristics of TCM, which provides a new idea for research into the effective substances and action mechanism of TCM [23]. Nonetheless, network analysis ignores whether the ingredients can be absorbed into the blood and its metabolism to have a curative effect, which may lead to unrealistic results [24,25]. Therefore, a new strategy was proposed in this study by combining integrated pharmacokinetics with network pharmacology, as well as considering the parameter (in vivo absorbed exposure), trying to identify potential active ingredients and clarify the in vivo mechanism of potential liver injury due to PM.

**Figure 1:**
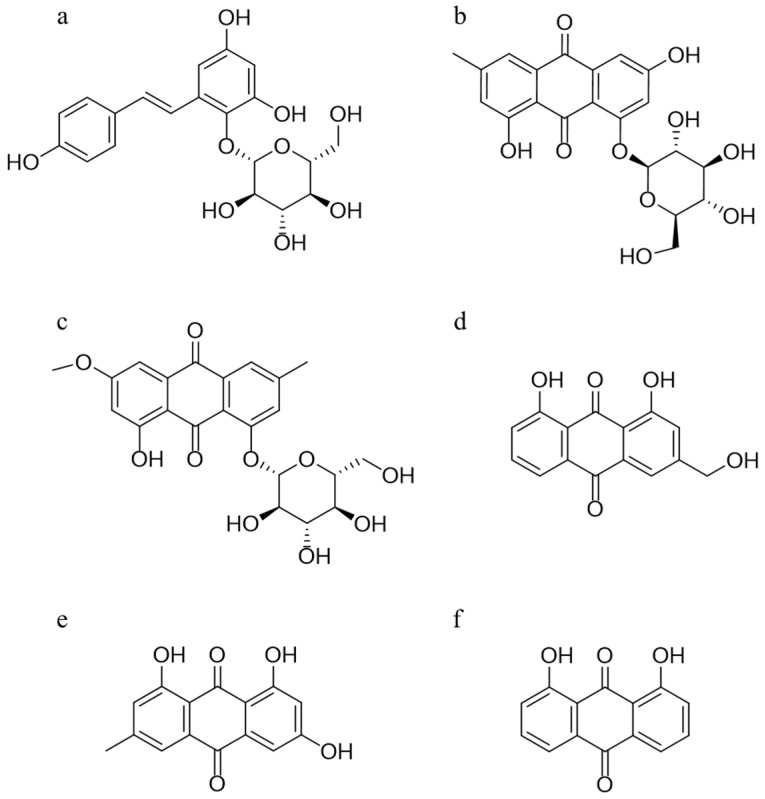
Chemical structures of TSG (**a**), EG (**b**), PG (**c**), AE (**d**), EM (**e**), and the internal standard 1,8-dihydroxyanthraquinone (**f**).

In this study, a rapid and sensitive HPLC-QQQ-MS method was established and well validated, which was applied to determine the serum level of five absorbed ingredients, trans-2,3,5,4′-tetrahydroxystilbene-2-*O*-β-d-glucoside (TSG), emodin-8-O-β-d-glucoside (EG), physcion-8-O-β-d-glucoside (PG), aloe-emodin (AE), and emodin (EM), after oral administration of PM extracts to Sprague–Dawley (SD) rats. The structures of these five compounds and 1, 8-dihydroxyanthraquinone (internal standard, IS) are given in Figure 1. A subsequent pharmacokinetics study was conducted to obtain the relevant pharmacokinetic parameters, including the mean concentration–time profiles. Next, integrated pharmacokinetics was used to obtain the integrated parameters. Finally, network pharmacology was carried out to perform the interaction between the five absorbed compounds and their targets, as well as the possible binding configurations and binding modes. The integrated pharmacokinetics-based HPLC-QQQ-MS method combined with network pharmacology was demonstrated to be a reliable approach for verifying the potential active components of PM, as well as clarifying their mechanism of potential liver injury.

## 2. Results

### 2.1. Method Validation

#### 2.1.1. Specificity

**Figure 2:**
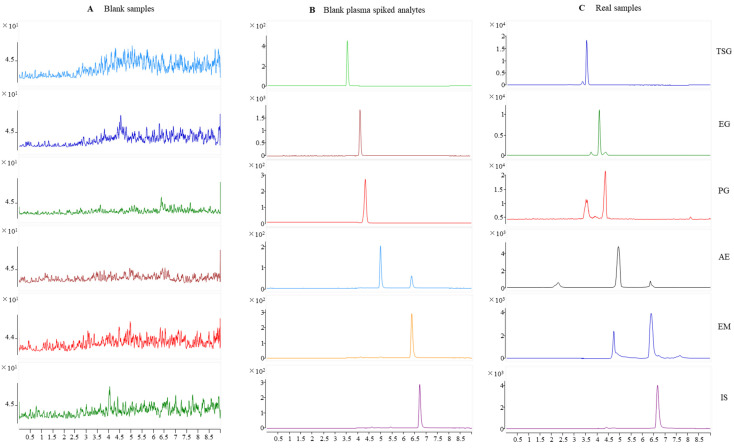
Representative MRM chromatograms for TSG, EG, PG, AE, EM, and IS in rat plasma. (**A**) Blank rat plasma; (**B**) blank rat plasma spiked with TSG, EG, PG, AE, EM, and IS; and (**C**) rat plasma samples after oral administration of PM for 30 min.

Typical chromatograms obtained from SD rat plasma samples are shown in Figure 2. Compared with a chromatogram of blank rat plasma, the endogenous components did not interfere with the TSG, EG, PG, AE, EM, and internal standard (IS) peaks. Specificity was found visibly for this method.

#### 2.1.2. Linearity

The regression equation, linear ranges correlation coefficient (r), and LLOQ are presented in Table 1. The results demonstrated a linearity of 0.500–800 ng/mL for TSG, PG, AE, and EM and 0.125~200 ng/mL for EG. The coefficient of correlation of all the calibration curves was more than 0.9951. The LLOQ of TSG, EG, PG, AE, and EM was 0.500, 0.125, 0.500, 0.500, and 0.500 ng/mL, respectively, which was appropriate for the quantification of the five analytes of plasma samples in the targeted pharmacokinetic study.

#### 2.1.3. Precision and Accuracy

As shown in Table 2, the intra- and inter-day precision and accuracy of the method were summarized. The intra- and inter-day precision of samples was within 18.6%, and the intra- and inter-day accuracy of these constituents ranged from 87.64% to 105.6%, respectively. The results indicated that the precision and accuracy are acceptable and the method is reliable.

#### 2.1.4. Extraction Recovery and Matrix Effect

The extraction recovery and matrix effect are shown in Table 3. For TSG, EG, PG, AE, and EM, these ranged from 85.36% to 111.5% and from 86.50% to 107.3%, respectively, and the RSD was less than 12.6% and 10.3%, respectively. The values indicated that there was no significant suppression or enhancement of ionization for the analytes.

#### 2.1.5. Stability

The results of short-term stability and long-term stability are presented in Table 4. The RSD of the values’ test responses were within 13.7% in all stability tests. Results demonstrated that TSG, EG, PG, AE, and EM are all stable in situations mimicking those encountered during sample storage, handling, and analysis (at 4 °C for 48 h and at −80 °C for 10 days, during three freeze–thaw cycles). No significant degradation was observed, and plasma samples processed under all the tested conditions were stable.

### 2.2. Compound Profile of PM

**Figure 3:**
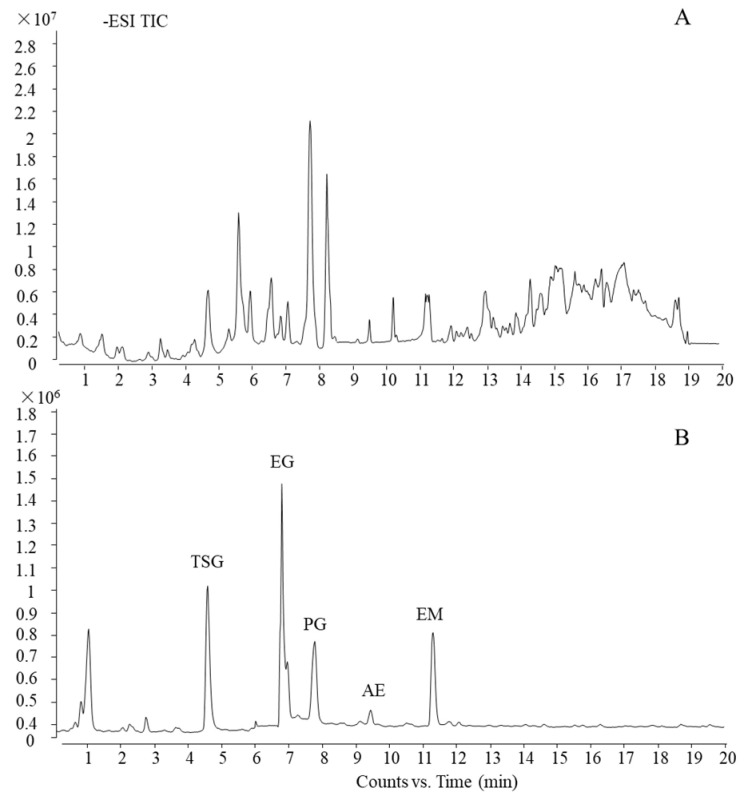
UHPLC-QTOF-MS profile of PM. (**A**) TIC of PM and (**B**) TIC of standard substances.

Using the current ultra-HPLC-quadrupole time-of-flight (UHPLC-QTOF)-MS method, which was reported in our previous work [26], the main five components of PM were characterized and confirmed by a standard substance as trans-2,3,5,4′-tetrahydroxystilbene-2-*O*-β-d-glucoside, emodin-8-O-β-d-glucoside, physcion-8-O-β-d-glucoside, aloe-emodin, and emodin (TSG, EG, PG, AE, and EM, respectively). The total ion chromatogram (TIC) of PM and standard substances is shown in Figure 3.

### 2.3. Integrated Pharmacokinetics

**Figure 4:**
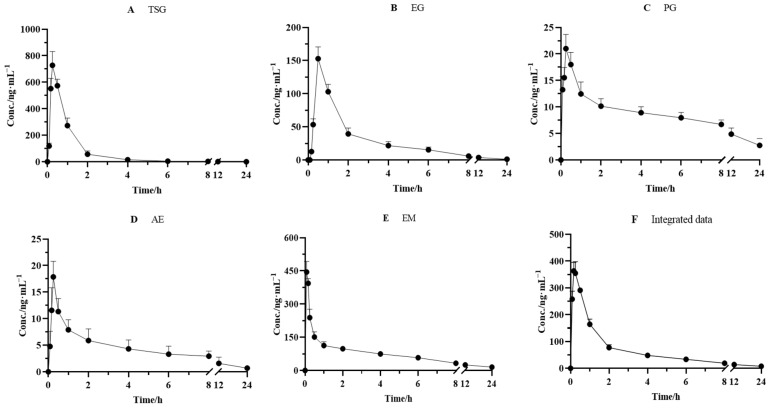
Mean plasma concentration–time curves for (**A**) TSG, (**B**) EG, (**C**) PG, (**D**) AE, and (**E**) EM and (**F**) integrated mean plasma concentration–time curve in rats after oral administration of PM (*n* = 6).

The validated method was successfully applied to the pharmacokinetic studies of the five effective components after the oral administration of PM (18 g/kg). The mean plasma concentration–time curves of TSG, EG, PG, AE, and EM are depicted in Figure 4. The pharmacokinetic and integrated pharmacokinetic parameters of TSG, EG, PG, AE, and EM were determined using DAS 3.2.8 software, and the calculated parameters are summarized in Table 5.

The plasma concentrations of the five components all increased rapidly to peak levels after oral administration. The T_max_ values of TSG, EG, PG, AE, and EM were 0.25 ± 0.00, 0.50 ± 0.00, 0.25 ± 0.00, 0.25 ± 0.0, and 0.17 ± 0.00 h, respectively, which indicated that the absorbance velocity of these compounds is relatively rapid and they may be quickly transported to the target site after entering the blood circulation system. We observed that TSG reached the highest C_max_ (728.0 ± 104.0 ng/mL) among the five constituents, followed by EM, with a C_max_ value of 388.2 ± 32.06 ng/mL. The C_max_ of EG, PG, and AE was 152.8 ± 17.97, 20.01 ± 2.692, and 17.86 ± 2.940 ng/mL, respectively. Moreover, the highest AUC_0–∞_, another PK parameter reflecting the levels of systemic exposure, was found for EM, reaching 1041 ± 300.2 ng·h/mL. Next came TSG, whose value reached 758.2 ± 58.60 ng·h/mL. The AUC_0–∞_ values of all other compounds ranged from 70.28 ± 13.85 ng·h/mL to 345.8 ± 48.45 ng·h/mL. In addition, the T_1/2_ value of TSG, EG, PG, AE, and EM was 2.22 ± 1.34, 6.47 ± 1.91, 12.3 ± 10.1, 6.42 ± 2.17, and 11.1 ± 5.22 h, respectively, which indicated that PG and EM had been eliminated relatively slowly.

It has been well acknowledged that the constitution of herbal medicine is highly complicated, and a single component’s pharmacokinetics alone cannot represent the medicine’s entire pharmacokinetic behavior. Considering the difference in pharmacokinetic parameters among TSG, EG, PG, AE, and EM, an AUC-weighting approach was applied to describe the holistic pharmacokinetic profiles of the five compounds. The weighting coefficients of TSG, EG, PG, AE, and EM were calculated using a formula (30.19%, 13.77%, 3.808%, 2.799%, and 49.43%, respectively). Next, we calculated the integral concentration according to the weight coefficient of each component to obtain the integrated drug–time curve, as shown in Figure 4, and its pharmacokinetic parameters are presented in Table 5. The results showed that the integrated pharmacokinetic parameters were as follows: T_max_ was 0.19 ± 0.041 h, C_max_ was 368.6 ± 33.37 ng/mL, AUC_0–∞_ was 914.7 ± 126.5 ng·h/mL, and T_1/2_ was 9.09 ± 4.05 h.

### 2.4. Compound Target Liver Injury Network Analysis

**Figure 5:**
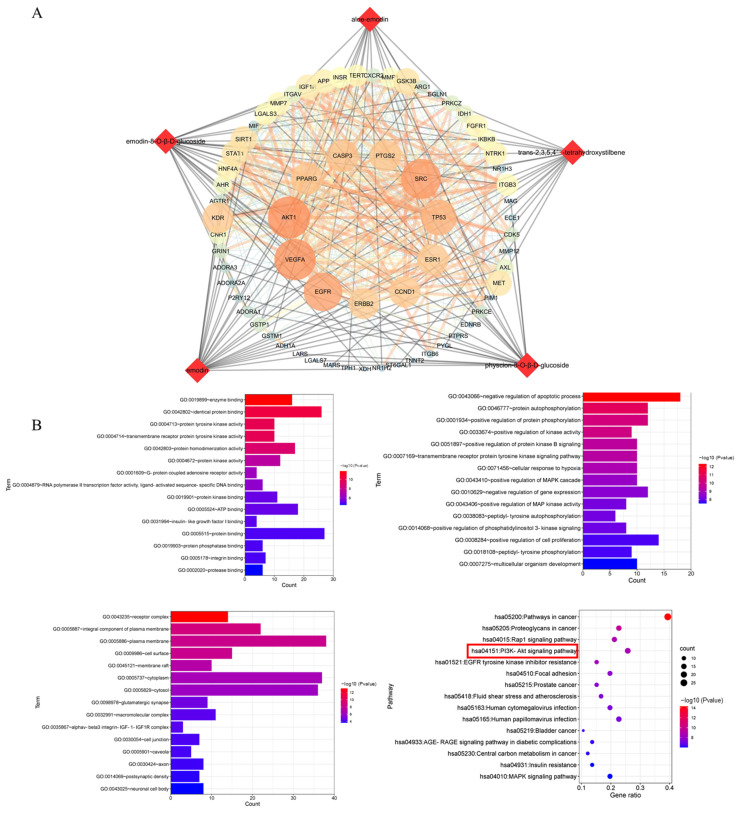
Network analysis of the five compounds absorbed into the blood after treatment with PM. (**A**) Compound target and liver injury target networks. Round nodes represent targets; red diamond nodes represent compounds. (**B**) GO analysis includes biological process and enriched KEGG pathways. The red box indicated the pathway most likely to be closely associated with liver injury.

According to the predicted results of the potential targets of the five absorbed compounds, 437 targets were screened from the Swiss Target and Pharm Mapper. A total of 655 targets were obtained from the databases of OMIM, which were related to potential liver injury. The STRING database exhibited the PPI network data of composite prediction targets and liver injury targets. Next, we obtained the compound target liver injury network using the merge function in Cytoscape software. As a result, 66 common targets of compound target liver injury networks were found through this network. Among them, AKT1 was the most important target with strong correlations (Figure 5A), followed by the DAVID database signaling-pathway-enriched common targets. We noticed that most of the pathways associated with potential liver injury were the PI3K-Akt signaling pathway, the MAPK signaling pathway, the Rap1 signaling pathway, and the Ras signaling pathway. In addition, it was found that the PI3K-Akt signaling pathway had a strong correlation with potential liver injury based on the *p*-value sequencing results (Figure 5B).

### 2.5. Verification by Molecular Docking

**Figure 6:**
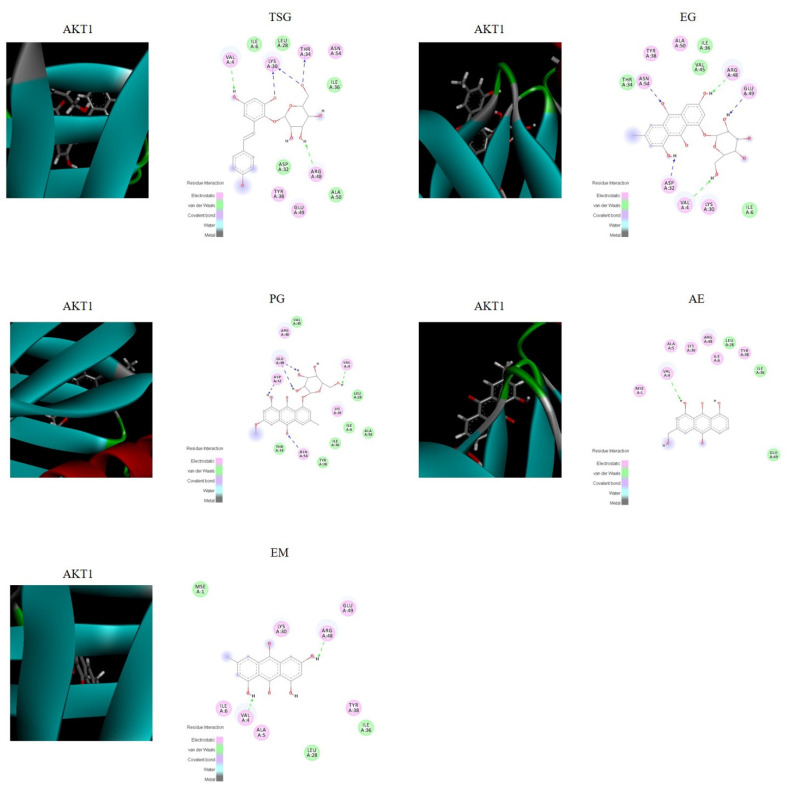
Visualization of the binding of hemostatic components to coagulation target protein (AKT1).

The molecular docking score is used to assess the potential toxicity of targeted molecules, based on the theory that a higher score usually represents a more toxic molecule; meanwhile, a higher total score means more stable ligand–target binding. As shown in Figure 6, the molecular docking results showed that AKT1 was well bound with all five absorbed compounds in vivo, with a total score above 4 (as shown in Table 6), and EG and PG showed strong binding, with a total score above 6. These results proved that these compounds absorbed into the blood have the potential to cause liver injury from the perspective of molecular docking.

## 3. Discussion

In this study, a strategy based on integrated pharmacokinetics and network pharmacology was established to investigate the potential liver injury mechanism of PM. A rapid and highly sensitive HPLC-QQQ-MS method was established for the pharmacokinetic study of the plasma of SD rats after oral administration of PM. At the same time, network pharmacology was used to explore the mechanism of absorption of components in vivo, and molecular docking was used to verify the results. Based on the research strategy, a feasible method reference was provided for revealing the potential liver injury mechanism of PM and provide scientific data support for the rational drug use of PM. In a broader sense, this combined strategy may also provide a reference for the study of related mechanisms of TCM [27].

The simultaneous quantification method was carried out via triple quadrupole–tandem mass spectrometry for the determination of the components (TSG, EG, PG, AE, and EM) of PM absorbed in vivo. The established method was rapid, sensitive, and efficient and could achieve the simultaneous quantitative analysis of the target compounds (five components and one IS) within 9 min. In addition, this method was verified and met the needs of sample analysis and determination in specificity, linearity, accuracy, precision, extraction recovery, matrix effect, and stability. We also optimized the method while the method was being established, including mass spectrum parameters and liquid-phase conditions. MS detection was performed in negative ion mode, which was more sensitive than in positive mode, for the five detected compounds. Our experimental results were in line with literature reports. Fragmentor voltage and CE were continually optimized for good MRM transitions of the five analytes. Chromatographic conditions were also optimized to suit the preclinical pharmacokinetic studies in our study. The peak shape improved by optimization of chromatographic conditions (buffer, mobile phase composition, and analytical column), increasing the signal intensity of the analytes. The mobile phase systems of water (A) and acetonitrile (B) and water (A) and methanol (B) at different flow rates were tested. Furthermore, to obtain higher sensitivity and a good peak shape, we also compared 0.1% formic acid in water and 0.1% formic acid in acetonitrile. The results showed that the responses of the analyte with acetonitrile and water as the mobile phase were obviously higher than those with methanol and water, and the addition of formic acid showed no significant improvement. Above all, an elution system (acetonitrile–water) was eventually determined to be the best mobile phase combination for the analytes at a flow rate of 0.6 mL/mL at 40 °C. To achieve better resolution, different columns were tested and the Agilent Poroshell 120 EC-C18 column (3.0 × 50 mm, 2.7 μm) was chosen for better separation.

The number of reports on the adverse effects of PM is increasing. Some researchers have found that PM may cause hepatotoxicity in long-term or high-dose use in clinic [28]. It has also been suggested that some specific genes are factors of PM-induced idiosyncratic liver injury [29]. Studies have revealed that that PM-associated liver damage can occur with no gender orientation and in any age group [30]. In most cases, the symptoms of liver damage occur about 1 month after taking the medicine, and they include fatigue, jaundice, anorexia, and yellow or tawny urine. A handful of patients were found with abdominal distension, abdominal pain, diarrhea, rash, pruritus, and other symptoms. After admission examination, a few cases were found with epigastrium tenderness, the first percussion over the liver, hepatomegaly or splenomegaly, and even ascites [31,32,33]. Nine case series reported the liver damage types in 221 patients, including 132 (132/221, 59.7%) patients with hepatocyte-type, 34 (34/221, 15.4%) patients with cholestatic-type, and 55 (55/221, 24.9%) patients with mixed-type liver damage [30]. In addition, laboratory animal studies have shown that PM has potential hepatotoxicity. Yang et al. revealed that a 70% ethanolic extract of PM induces considerably higher liver toxicity in zebrafish than other solvent extracts of PM, such as water, acetone, methanol, and a lower percentage of ethanol [34]. The oral administration of 95% ethanol extracts of PM to male SD rats in three groups (19.2, 192, and 1920 mg/kg/d) for 28 days showed increased levels of ALT, AST, and ASP, together with reduced activity of SOD, indicating higher liver damage in the rats taking a medium and a high dose of PM [35]. Another study established the dose–time–toxicity relationship of the hepatotoxicity caused by administration of a single dose of the water-extracted and ethanol-extracted components of PM to mice. The water-extracted components (from 5.5 to 30.75 g/kg) and the ethanol-extracted components (from 8.5 to 24.5 g/kg) caused obvious damage to the liver organization, resulting in significantly increased serum ALT and AST levels, and this effect was dose dependent [36]. All these findings confirm that PM has potential hepatotoxicity.

The exact pharmacokinetic characteristics of a single component can be obtained by traditional pharmacokinetic studies, but the isolated pharmacokinetic behavior of each component is not enough to comprehensively characterize the overall pharmacokinetic characteristics of TCM. Furthermore, an integrated pharmacokinetic study was performed on the components of PM for the first time in order to conform the characteristics of the multiple components and multiple targets of TCM. The plasma pharmacokinetic parameters of the constituents were different, as summarized in Table 5. The AUC_0→∞_ of the integrated pharmacokinetic parameters was 914.7 ± 126.5 ng h/mL, which is due to the weighting coefficient of each component, indicating that compounds EM and TSG accounted for the most weight and contributed the most to the whole pharmacokinetic parameters (49.43% and 30.19%, respectively). This is related to the high absorption into the body (AUC_0→∞_ of EM and TSG was 1024 ± 300.2 and 758.2 ± 58.60 ng h/mL, respectively). TSG, EG, and AE have shorter half-lives (T_1/2_); PG and EM have relatively long half-lives (12.31 ± 10.09 and 11.09 ± 5.219 h, respectively). The half-life after integration was 9.09 ± 4.05 h, and it was seen that PG and EM contributed a large proportion. According to the half-life (T_1/2_), the clearance rate (CLz/F), and the average residence time (MRT), the overall component could still be detected at 24 h after administration, elimination was slow, and it easily persisted in the body, suggesting that this may be related to the potential liver injury due to PM. Literature reports have shown that TSG, EM, and EG may be the material basis of PM-induced specific liver injury, and they have synergistic effects [37,38,39,40]. In our integrated pharmacokinetic study, the weights of TSG, EM, and EG were 30.19%, 49.43%, and 13.77%, respectively, and the sum of the three was 93.39%, accounting for a relatively large proportion, which provided a reference for the above theory at the level of substance content in vivo.

The integrated pharmacokinetic results were correlated with the pharmacodynamic results [17]. In this study, we conducted a network pharmacological study based on the real chemical components of PM that are absorbed into the body, which effectively avoided the inaccurate results caused by the literature research only. Pathway enrichment results showed that the PI3K-Akt signaling pathway, the MAPK signaling pathway, the Rap1 signaling pathway, and the Ras signaling pathway are strongly correlated with liver injury; PI3K/Akt has a strong correlation. Akt, a serine/threonine kinase, is an important protein in the PI3K pathway and plays a key role in cell physiological processes, including cell glucose metabolism, cell proliferation, cell migration, and cell apoptosis [41]. There are three Akt isoforms: PKBα (Akt1), PKBβ (Akt2), and PKBγ (Akt3). Akt1 is expressed in various tissues. Akt2 is mainly expressed in insulin-sensitive tissues, such as skeletal muscle, adipose tissue, and liver, and Akt3 is mainly expressed in the testes and brain [42,43]. The PI3K/Akt signaling pathway is closely related to hepatocyte inflammation, apoptosis, and oxidative stress [44]. Network pharmacological results showed that AKT1, REGF, SCR, and VEGFA are important targets with a strong correlation, their common targets are enriched by database signaling pathways, and AKT1 is the most correlated target. The results of molecular docking showed that these five intracellular components are indeed closely related to the AKT1 target (scores greater than 6 or 4), which confirmed the reliability of the method strategy. To the best of our knowledge, this is the first study to combine the in vivo composition of PM (considering the in vivo pharmacokinetic parameters) with network pharmacology.

## 4. Materials and Methods

### 4.1. Chemicals and Reagents

TSG (purity 98.0%), EG (purity ≥ 98.0%), PG (purity ≥ 95.0%), AE (purity ≥ 95.0%), and IS (purity ≥ 98.0%) were purchased from Chengdu Herbpurify CO., Ltd. (Chengdu, China). EM (purity ≥ 98.0%) was obtained from Nantong Feiyu Biological Technology Co., Ltd. (Jiangsu, China). Dimethyl sulfoxide (DMSO) (MS grade) was obtained from Sigma-Aldrich (St. Louis, MO, USA). The structures of all the standards are shown in Figure 1. Acetonitrile and methanol (LC/MS grade) were purchased from Merck Company (Darmstadt, Germany). Water was obtained using a Milli Q system (Millipore, Bedford, MA, USA).

PM was purchased from Beijing San He Co., Ltd. (Beijing, China) and authenticated by Prof. Xueyong Wang. The voucher specimen (CMAT-PM-201901) has been deposited at the Research Centre for Chinese Medical Analysis and Transformation, Beijing University of Chinese Medicine (BUCM, Beijing, China).

### 4.2. PM Preparation

The extraction of PM extract has been reported in our previous article [5]. Briefly, 43 kg of PM was extracted with 70% ethanol (430 L × 1.5 h) three times. A total of 5.4 kg powder was obtained through the extraction.

### 4.3. Animals

Adult male Sprague–Dawley (SD) rats weighing between 180 g and 220 g were purchased from Beijing Vital River Laboratory Animal Technology Co., Ltd. (Beijing, China). The rats were kept in an animal center with air-conditioning and with a natural light–dark cycle (22 ± 1 °C and 40–50% humidity) with ad libitum access to standard food and water for a week before the experiment. All the animal procedures were in accordance with the Regulations of Experimental Animal Administration issued by the State Committee of Science and Technology of the People’s Republic of China. All the SD rats were fasted for 12 h prior to the experiment but with free access to water.

### 4.4. Instrumentation and HPLC-QQQ-MS Conditions

The assay was performed with an Agilent 1260 high-performance liquid chromatography system combined with Agilent 6470 QQQ MS (Agilent Technologies, CA, USA). Applied Masshunter Qualitative Analysis software (version B.07.00) and Quantitative Analysis software (version 07.00) were used for data acquisition and quantification. Analytes separation was achieved on a reversed-phase C18 column (Agilent Poroshell 120 EC-C18, 3.0 × 50 mm, 2.7 μm), which was protected by a guard column (Agilent EC-C18 3.0 × 5.0 mm, 2.7 μm). A gradient elution program composed of water (A) and acetonitrile (B) with gradient elution was used as follows: 0–1 min, 5% B; 1–3 min, 5–45% B; 3–7 min, 45–60% B; and 7–9 min, 60–95% B. The oven temperature was maintained at 40 °C. The flow rate was set at 0.6 mL/min (with a split ratio of 1:1.8), and the injection volume was 4 μL.

Agilent QQQ MS equipped with an electrospray ionization (ESI) source was used for mass spectrometric detection. MRM mode (negative) was used for performing the quantification of TSG, EG, PG, AE, and EM. MS parameters for each analyte and IS are shown in Table 7. The other MS conditions were set as follows: gas temperature, 300 °C, gas flow, 8 L/min; nebulizer, 55 psi; and sheath gas flow, 11 L/min.

### 4.5. Preparation of Standard Solutions and Quality Control (QC) Samples

IS stock solution was prepared with a concentration of 2.0 μg/mL using methanol and then diluted to 100 ng/mL to obtain IS solution. The stock solutions of TSG, EG, PG, AE, and EM that were used to make the calibration standards were prepared by dissolving 5 mg of each compound in 5.0 mL of the solvent (containing 500 μL of DMSO and 4.5 mL of methanol) to obtain a concentration of 1.00 mg/mL of each compound. Serial standard working solutions with different concentrations were prepared through blends and dilutions of the stock solutions with methanol. The calibration standard solutions containing eight different concentrations (TSG, PG, AE, and EM: 0.500, 1.00, 2.00, 10.0, 20.0, 100, 200, 400, and 800 ng/mL; EG: 0.125, 0.25, 0.50, 2.50, 5.00, 25.0, 50.0, 100, and 200 ng/mL) were prepared by spiking blank rat plasma and IS solution (100 ng/mL) with appropriate concentrations of TSG, EG, PG, AE, and EM.

Samples for bioanalytical method validation were prepared by spiking 100 μL of blank rat plasma and 100 μL of IS solution in bulk with different standard solutions to obtain appropriate concentrations for serial standard working solutions with the matrix and QC samples (TSG, PG, AE, and EM: 0.50 ng/mL (lower limit of quantitation, LLOQ), 10.0 ng/mL (low quality control, LQC), 200 ng/mL (medium quality control, MQC), and 640 ng/mL (high quality control, HQC); EG: 0.125 ng/mL (LLOQ), 2.50 ng/mL (LQC), 50.0 ng/mL (MQC), and 160 ng/mL (HQC)). All the stock solutions, working solutions, and samples were stored at −80 °C pending use.

### 4.6. Plasma Sample Preparation

A simple pretreatment method for protein precipitation was carried out to clean up the plasma samples prior to LC-MS/MS analysis. The plasma sample was thawed to room temperature. An aliquot of pre-cooled 800 μL of acetonitrile and 100 μL of IS solution (100 ng/mL) was added to a 300 μL plasma sample in a centrifuge tube. The tubes were vortexed for 10 min and spun in a centrifuge at 12,000 rpm for 15 min at 4 °C. The supernatant was transferred into a separate tube and dried using a vacuum concentrator, and the samples were re-dissolved with 100 μL of pure methanol. Next, the tube was vortexed for 10 min and spun in a centrifuge at 12,000 rpm for 15 min at 4 °C again. The supernatant was then injected into the HPLC-QQQ-MS instrument for analysis.

### 4.7. Bioanalytical Method Validation

The established method was well validated before sample analysis. Specificity, linearity, precision, accuracy, recovery, matrix effect, and stability were validated according to the United States Food and Drug Administration (US FDA) bioanalytical method validation guidance [45].

#### 4.7.1. Specificity

Specificity was determined by analysis of at least 6 individual blank rat plasma samples, and every blank sample was handled by the procedure described in Section 4.6 to ensure that endogenous substances would have no possible interference with the five analytes and the IS.

#### 4.7.2. Linearity and Lower Limit of Quantitation (LLOQ)

Matrix-matched calibration standard solutions of eight concentration levels were prepared, as described before. The linearity of each calibration curve was constructed by plotting peak area ratios (y) of three constituents to the IS versus respective plasma concentrations (x) using a 1/x2 weighted linear least squares regression. The LLOQ for the analytes was the lowest concentration of the drug in spiked plasma on the calibration curve resulting in a signal-to-noise (S/N) ratio greater than 10. The LOQ was measured with an accuracy of 80–120% and precision less than 20%, while other concentrations had an accuracy of 85–115% and precision less than 15%.

#### 4.7.3. Precision and Accuracy

Precision and accuracy were evaluated by analyzing six replicate QC samples at the LQC, MQC, and HQC of all analytes. The intra-day precision and accuracy were evaluated within 1 day by analyzing six replicates at each concentration level. The inter-day precision and accuracy were investigated on 3 successive days by repeating the process. The relative error (RE), used to express accuracy, should range from 85 to 115%, while the relative standard deviation (RSD) selected for intra-/inter-day precision assessment should not exceed 15%.

#### 4.7.4. Extraction Recovery and Matrix Effect

For the extraction recovery of the detected compounds, three levels of QC were obtained by comparing the peak areas of analytes in the plasma samples with the peak areas of the same analytes spiked after and before extraction. The matrix effects were measured by comparing the peak areas of the analytes in the spiked postextraction samples with those of the same analytes at the same concentrations dissolved in methanol. Six replicates were performed of all QC samples.

#### 4.7.5. Stability

Stability experiments were performed to evaluate the stability of the analytes in rat plasma at the LQC, MQC, and HQC under different time and temperature conditions. These included short-term stability (4 °C for 48 h), long-term stability (−80 °C for 10 days), and stability over three freeze–thaw cycles (−80 °C to 25 °C) by analyzing QC samples.

### 4.8. Integrated Pharmacokinetic Application

Blood samples were collected from the fundus into heparinized 1.5 mL polythene tubes at 5, 15, and 30 min and 1, 2, 4, 8, 12, and 24 h after oral administration of PM (18 g/kg). The blood samples were immediately centrifuged at 3500 rpm for 10 min to obtain plasma. Next, the plasma obtained was prepared for HPLC-QQQ-MS analysis, as described before. The TSG, EG, PG, AE, and EM concentrations in plasma versus time data for each rat were analyzed using Drug and Statistics Software (DAS version 3.2.8, Beijing University of Traditional Chinese Medicine, Beijing, China).

As mentioned before, the concentration–time curve (AUC_0–∞_) of each constituent was obtained using DAS, and then the integrated concentration was obtain using the following equations [46]: The weighting coefficient for each component was calculated using Equations (1) and (2). The integrated concentrations were then calculated by Equation (3).

(1) $$W_{j}=\frac{{\mathrm{AUC}}_{j0-\infty }}{\sum_{1}^{3}{\mathrm{AUC}}_{0-\infty }}$$

(2) $$\overset{3}{\underset{1}{\sum }}{\mathrm{AUC}}_{0-\infty }={\mathrm{AUC}}_{0-\infty 1}+{\mathrm{AUC}}_{0-\infty 2}+{\mathrm{AUC}}_{0-\infty 3}$$

(3) $$c_{T}=W_{1}\times c_{1}+W_{2}\times c_{2}+W_{3}\times c_{3}$$

where ω represents the weighting coefficient, “j” represents the three constituents studied, C1–C3 represent the plasma concentration of the three index components studied, and CT represents the integrated plasma concentration.

### 4.9. Network Pharmacology

The network construction mainly included the following four steps:

(1) Prediction of the potential targets of five absorbed compounds. Canonical SMILES format of the five compounds in vivo were converted through the PubChem database. Next, the Swiss bioinformatics research Target Prediction database (http://www.Swisstargetprediction.ch/, accessed on 19 October 2022) and Pharm Mapper (http://www.lilab-ecust.cn/pharmmapper/, accessed on 19 October 2022) were used for the prediction of potential liver injury targets.

(2) Collection of the target protein. We searched the keywords (such as “liver injury”, “toxicity of liver”, and “hepatoxicity”) in the OMIM database (https://omim.org/, accessed on 19 October 2022), obtaining the targets related to liver injury.

(3) Construction and analysis of biological networks. The online database STRING (https://string-db.org/, accessed on 19 October 2022) could provide information about the interaction and predictive role of proteins [47]. The selected proteins were imported and the species *Homo sapiens* was selected. Next, the relevant protein–protein interaction (PPI) data that evaluate and assign the information about each protein interaction were obtained. Cytoscape 3.7.1 software was used to visualize the PPI data; the degree and closeness centrality of network topology parameters were used to analyze the targets in the network.

(4) Enrichment analysis. The cross-set subnetwork between PM and the liver injury network was extracted, and the target with a median value higher than the average value of the subnetwork was considered as an important target. The key target was selected by the intersection of the important targets and the important modules. Next, functional enrichment analysis was performed through the DAVID database (https://david.ncifcrf.gov/home.jsp, accessed on 19 October 2022) and Gene Ontology (GO); meanwhile, pathway enrichment analysis was carried out using the Kyoto Encyclopedia of Genes and Genomes (KEGG) database.

The five effective compounds were docked with target proteins using SYBYL-X software (version 2.0). The target protein was docked with the chemical components of PM to verify the potential hepatotoxicity of the underlying components. Toxicity was assessed based on the docking score, meaning that the higher the score, the more active the compound. The threshold was set as 5, and scores of molecules above the threshold were considered to cause potential liver damage [48] Through this method, the results of network pharmacology were verified, and the five compounds studied by pharmacokinetics were illustrated as components with the potential to cause liver injury.

## 5. Conclusions

In this study, a strategy combining network pharmacology with integrated pharmacokinetics was established to explore the pharmacological mechanism of potential liver injury due to PM. The established and well-validated HPLC-QQQ-MS methodology was proven to be suitable and competent for quantification of the five components (TSG, EG, PG, AE, and EM) and providing pharmacokinetic profiles. At the same time, the integrated pharmacokinetic parameters may help to better understand the in vivo mechanism of the effective compounds of TCM. Additionally, the network pharmacological study was considered a successful method for illustrating the potential liver injury mechanism of the absorbed components of PM, demonstrating that PI3K-Akt, MAPK, Rap1, and Ras signaling pathways may be the ways through which the absorbed compounds perform their functions. Moreover, among them, AKT1 was the most correlated target. This study could improve the safety and rationality of the clinical use of PM and, in a broader sense, provides a practical strategy to systematically explore the potential therapeutic/toxic mechanism of TCM, which is undoubtedly of great significance for its clinical application.

## Figures and Tables

**Table 1:** The results of linear ranges, regression equations, and LLOQs of five detected compounds.

Analytes	Linear Range (ng/mL)	Regression Equation	Correlation Coefficient (r)	LLOQ (ng/mL)
TSG	0.500~800	y = 0.7675x + 0.0034	0.9985	0.500
EG	0.125~200	y = 2.7659x + 0.0067	0.9976	0.125
PG	0.500~800	y = 8.2713x + 0.0198	0.9992	0.500
AE	0.500~800	y = 0.2659x + 0.0012	0.9951	0.500
EM	0.500~800	y = 0.6200x + 0.0700	0.9968	0.500

**Table 2:** Precision and accuracy of the method for the determination of TSG, EG, PG, AE, and EM in rat plasma (*n* = 6).

Analytes	Spiked Conc. (ng/mL)	Intra-Day		Inter-Day	
		RSD (%)	Re (%)	RSD (%)	Re (%)
TSG	10.0	11.0	96.81	6.59	89.83
	200	2.97	94.06	8.70	92.50
	640	5.06	92.81	5.85	88.73
EG	2.50	8.12	96.56	18.6	97.57
	50.0	4.43	95.66	10.9	105.6
	160	4.98	91.12	4.69	88.95
PG	10.0	6.99	100.2	15.6	87.64
	200	10.1	97.83	5.86	92.58
	640	4.37	98.976	9.23	95.36
AE	10.0	7.25	91.25	11.5	90.19
	200	7.39	88.99	5.76	88.12
	640	5.20	92.35	9.31	89.65
EM	10.0	5.97	94.00	12.4	97.56
	200	1.39	100.9	6.31	92.65
	640	6.26	92.84	10.6	98.39

**Table 3:** Recovery and matrix effect of TSG, EG, PG, AE, and EM (*n* = 6).

Analytes	Spiked Conc. (ng/mL)	Extraction Recovery		Matrix Effect	
		RSD (%)	Re (%)	RSD (%)	Re (%)
TSG	10.0	11.4	107.8	8.58	99.00
	200	4.89	103.5	6.29	94.40
	640	4.76	97.73	1.29	86.50
EG	2.50	11.9	102.8	10.1	99.30
	50.0	2.72	99.31	8.80	100.5
	160	4.30	97.79	4.16	92.00
PG	10.0	12.6	95.61	8.46	87.61
	200	9.88	91.38	9.28	95.38
	640	7.69	99.01	8.72	94.27
AE	10.0	9.17	85.36	10.3	98.72
	200	6.52	90.31	7.90	90.19
	640	3.28	95.27	6.92	96.97
EM	10.0	5.83	99.64	1.71	94.50
	200	2.77	111.5	6.09	107.3
	640	2.32	105.7	4.17	90.70

**Table 4:** Stability results of TSG, EG, PG, AE, and EM in rat plasma under different conditions (*n* = 6).

Analytes	Spiked Conc. (ng/mL)	4 °C, 48 h		−80 °C, 10 Days		Three Freeze–Thaw Cycles	
		RSD (%)	Re (%)	RSD (%)	Re (%)	RSD (%)	Re (%)
TSG	10.0	3.50	100.1	5.46	97.92	7.38	91.38
	200	8.40	92.13	1.77	95.95	3.69	95.27
	640	3.16	97.86	3.70	92.72	3.27	99.01
EG	2.50	3.84	104.2	4.50	105.4	9.88	94.39
	50.0	11.9	93.92	2.90	103.6	5.61	96.21
	160	4.43	94.28	1.62	96.66	5.09	91.37
PG	10.0	13.7	98.55	12.7	90.14	10.9	88.21
	200	5.28	90.36	9.81	94.69	6.90	94.08
	640	9.29	92.41	9.95	96.88	5.24	98.03
AE	10.0	5.39	94.77	6.28	90.27	4.33	90.50
	200	7.77	97.05	8.21	89.62	4.29	91.44
	640	8.91	90.21	7.76	100.1	6.18	96.94
EM	10.0	7.16	86.36	2.20	97.48	8.27	96.37
	200	6.25	92.05	1.38	99.40	4.14	98.71
	640	9.50	85.19	2.14	111.7	4.53	99.10

**Table 5:** Pharmacokinetic and integral pharmacokinetics parameters of PM using an AUC-based weighting approach (*n* = 6).

Parameters	TSG	EG	PG	AE	EM	Integrated Data
T_1/2z_ (h)	2.22 ± 1.34	6.47 ± 1.91	12.3 ± 10.1	6.42 ± 2.17	11.1 ± 5.22	9.09 ± 4.05
C_max_ (ng/mL)	728.0 ± 104.0	152.8 ± 17.97	20.01 ± 2.692	17.86 ± 2.940	388.2 ± 32.06	368.6 ± 33.37
T_max_ (h)	0.25 ± 0.00	0.50 ± 0.00	0.25 ± 0.00	0.25 ± 0.00	0.17 ± 0.00	0.19 ± 0.041
AUC_0–t_ (ng h/mL)	757.7 ± 58.88	333.5 ± 39.74	146.3 ± 19.40	64.54 ± 9.397	1021 ± 142.3	833.0 ± 77.63
AUC_0–∞_ (ng h/mL)	758.2 ± 58.60	345.8 ± 48.45	205.0 ± 95.62	70.28 ± 13.85	1041 ± 300.2	914.7 ± 126.5
MRT_0–t_ (h)	1.215 ± 0.1820	3.735 ± 0.5270	8.415 ± 1.015	6.503 ± 1.423	6.822 ± 0.5450	4.958 ± 0.4720
MRT_0–∞_ (h)	1.232 ± 0.2030	4.759 ± 1.224	17.65 ± 13.90	8.574 ± 3.019	12.71 ± 5.092	7.955 ± 2.969
Vz/F (L/kg)	2005 ± 1274	379.1 ± 95.41	242.1 ± 71.82	407.3 ± 113.3	152.1 ± 51.54	7024 ± 2524
CLz/F (kg L/h)	617.8 ± 46.15	41.30 ± 5.423	17.38 ± 6.193	45.36 ± 7.850	10.25 ± 2.540	554.1 ± 78.31

**Table 6:** Score of molecular docking.

Name	TSG	EG	PG	AE	EM
AKT1	4.84	6.68	6.54	4.46	4.01

**Table 7:** The optimized mass spectrometry parameters of the five constituents of RM and IS.

Analytes	Ion Mode	Transition	Fragmentor (V)	Collision Energy (V)
TSG	-	405.2→243.1	145	15
EG	-	431.1→269.1	190	30
PG	-	445.2→283.1	145	30
AE	-	269.1→240.1	135	25
EM	-	269.0→182.0	145	40
IS	-	239.0→210.8	145	30

## Data Availability

The authors declare that all data supporting the findings of this study are available within the article.

## Sample Availability

Samples of the compounds are available from the authors.
